# Post-coronavirus disease 2019–associated symptoms among children and adolescents in the SARS-CoV-2 Omicron era

**DOI:** 10.1007/s00431-024-05919-3

**Published:** 2024-12-21

**Authors:** Anne Schlegtendal, Christoph Maier, Julien Stein, Jakob Höpner, Astrid Petersmann, Denisa Drinka, Reinhard Berner, Thomas Lücke, Nicole Toepfner, Folke Brinkmann

**Affiliations:** 1 University Children´s Hospital, St. Josef-Hospital, Ruhr-University Bochum, Bochum, Germany; 2 Department of Medical Informatics, Biometry and Epidemiology, Ruhr-University Bochum, Bochum, Germany; 3 Institute for Clinical Chemistry and Laboratory Medicine, University Medicine Oldenburg, Oldenburg, Germany; 4 Institute for Clinical Chemistry and Laboratory Medicine, University Medicine Greifswald, Greifswald, Germany; 5 Faculty of Medicine and University Hospital Carl Gustav Carus, Department of Pediatrics, TUD Dresden University of Technology, Dresden, Germany; 6 Department of Pediatrics, Section of Pediatric Pneumology, University of Lübeck, Campus Lübeck, Lübeck, Germany; 7 Airway Research Center North (ARCN), Germany, Member of the German Center for Lung Research (DZL), Düsseldorf, Germany

**Keywords:** Post-COVID-19 syndrome (PCS), Long COVID, SARS-CoV-2, Omicron, Children and adolescents

## Abstract

**Purpose:**

Lack of a control group(s) and selection bias were the main criticisms of previous studies investigating the prevalence of post-coronavirus disease 2019 (COVID-19) syndrome (PCS). There are insufficient data regarding paediatric PCS, particularly in the SARS-CoV-2 Omicron era. As such, our study investigated PCS-associated symptoms in a representative control-matched cohort.

**Methods:**

This multicentre, cross-sectional, cohort study within the “Immunebridge” project of the German Network University Medicine (NUM) recruited children and adolescents (five to 17 years old) between July and October 2022. Children with polymerase chain reaction-confirmed SARS-CoV-2 infection in 2022 (COVID-19 group) were compared with those without history of SARS-CoV-2 infection and negative for SARS-CoV-2 antibodies. Queries included vaccinations, quality of life (QoL), and mental and physical symptoms potentially associated with PCS in the previous three months. An additional composite item, “physical performance”, was created from the responses.

**Results:**

The number of children with ≥ 1 PCS symptom(s) was comparable between the COVID-19 (*n* = 114 [62.1%]) and control (*n* = 66 [64.9%]) groups. Concentration disorders were reported more frequently in the COVID-19 group (12.3% versus 1.5%; *p* = 0.012) and “physical performance” was significantly impaired (*p* = 0.016) regardless of age, sex, and SARS-CoV-2 vaccination. The frequencies of other symptoms were similar in both groups. The COVID-19 group rated their fitness as worse, with otherwise equal QoL ratings regarding general and mental health.

*Conclusion* Children with and without previous infections did not differ in most PCS-associated symptoms. Exceptions included physical performance and cognitive problems, which appeared to be more impaired after Omicron infection than in controls.

**Supplementary information:**

The online version contains supplementary material available at 10.1007/s00431-024-05919-3.

**Table:** 

**What is Known:** • *Mainly due to too few controlled studies, knowledge about the prevalence of individual symptoms in paediatric post-COVID-19 syndrome (PCS) for the Omicron era is poor.*
**What is New:** • *In a representative control-matched cohort most parent-reported PCS-associated symptoms and quality of life scores in children and adolescents with PCR-confirmed SARS-CoV-2 infection did not differ from those without infection.* • *Exceptions were physical performance and cognitive problems, which were apparently more severely impaired after SARS-CoV-2-Omicron infection than in the control subjects.*

## Introduction and background

Long coronavirus disease-2019 (COVID-19 [“long COVID”]) or post-COVID-19 syndrome (PCS) is increasingly reported as a health problem not only in adults but also in children and adolescents after infection with severe acute respiratory syndromecoronavirus type 2 (SARS-CoV-2) [1, 2]. In February 2023, the World Health Organisation (WHO) defined PCS in children and adolescents as a condition occurring in “individuals with a history of confirmed or probable SARS-CoV-2 infection, when experiencing symptoms lasting at least 2 months which initially occurred within 3 months of acute COVID-19” [3]. According to the WHO, the most frequently reported symptoms of PCS include fatigue, altered smell/anosmia, and anxiety. In addition, many other―sometimes nonspecific―symptoms may occur in different organ systems, including chest pain, cognitive difficulties, dyspnoea, headache, abdominal pain, and sore eyes and throat [3]. These symptoms can impair everyday function(s) in affected children and adolescents. However, due to various disease definitions in the past and wide heterogeneity in the described symptoms and different study designs (such as variable follow-up times, reliance on self- or parent-reported symptoms without clinical assessment, lack of objective testing for SARS-CoV-2 immune status, and missing control group), data regarding the prevalence of PCS in children and adolescents are, in part, conflicting. A large meta-analysis published in June 2022, which included data from > 80,000 children from 21 studies, reported an overall PCS prevalence of 25.24% (95% confidence interval 18.17–33.02) [4]. In contrast, other studies that included negative control groups reported substantially lower prevalence rates, ranging from 1.8% to 4.6% [5–7].

During the “Omicron wave” (starting in January 2022), significantly more acute SARS-CoV-2 infections were reported in children and adolescents in Germany than in the earlier stages of the pandemic [8, 9], and the seroprevalence increased significantly [10–12]. Omicron infections in children are commonly reported to be asymptomatic or mild, and cases of multisystem inflammatory syndrome in children (i.e. “MIS-C”) occur much less often [8]. However, there is little supportive evidence to suggest that an increase in infections leads to a corresponding increase in PCS among children and adolescents. In adults, the prevalence of individuals with PCS symptoms decreased to 4.5% during the Omicron wave (compared with 10.8% during the Delta wave) [13], and adults appeared to experience fewer PCS symptoms compared with previous variants [14]. In contrast to the decreasing PCS symptom rates in adults, data regarding changes in symptom rates, profile, severity, and resulting frequency of PCS among children and adolescents during the Omicron era are partially contradictory [15–17]. As other studies have recently reported [18], those investigating PCS are often limited by the lack of a control group in general or by the lack of confirmed SARS-CoV-2 serostatus in the control group.

The aim of our study, therefore, was to investigate the frequency and severity of persistent PCS-associated symptoms in a non-selective cohort of children and adolescents after SARS-CoV-2 Omicron infection compared with an age-matched SARS-CoV-2 seronegative control group.

## Methods

As part of the “Immunebridge” project of the German Network University Medicine (NUM) [11, 19], between July and October 2022, children > 5 years of age and adolescents were recruited for a cross-sectional cohort study at study centres in Bochum and Dresden, Germany, providing tertiary medical care in two large German metropolitan areas, the Ruhr area in the west of Germany and the metropolitan area of the Saxon Triangle in the east of Germany. Adolescents or students, respectively, from classes in two secondary schools from grade 8 and onwards in Dresden were invited to participate as amendment to the SchoolCoviDD19 study from 2020 [20]. In Bochum, children and adolescents of a population-based cohort investigation performed in the Ruhr Region in 2020 and 2021 [21] were invited to participate in a follow-up study. Of the original 2184 participants, only families with current contact details living in the Bochum area and who had originally agreed to be addressed for a follow-up were contacted (details described in “Höppner et al.”, under review). Parents of 789 children and adolescents of this Bochum “Corkid” cohort were contacted, on average, after 2 years.

The minimum age for inclusion was set at five years to obtain reliable information about the participants’ physical and mental symptoms. The COVID-19 group comprised children and adolescents with a history of SARS-CoV-2 infection confirmed by a positive polymerase chain reaction (PCR) test after 1 January 2022, as reported by parents/participants in the study questionnaire. The control group comprised children of the same age with negative PCR results and nucleocapsid antibodies against SARS-CoV-2.

Antibodies against SARS-CoV-2 were identified in the blood samples of all study participants. Blood plasma samples were analysed directly in one laboratory. SARS-CoV-2 antibody determination was performed in all children and adolescents using commercially available immunological electrochemiluminescence assays (ECLIA, Roche Diagnostics GmbH, Mannheim, Germany). Antibodies (AK) against the SARS-CoV-2 spike (S) protein (S-AK) occurring after vaccination and/or illness were quantitatively assessed (positive, ≥ 0.8 binding antibody units [BAU]/ml). Antibodies against the nucleocapsid (N) protein (N-AK) specific for a previous infection were qualitatively assessed (positive, assay-specific cut-off index [COI], ≥ 1.0 COI).

Parents, children, and adolescents were asked to complete a self-administered questionnaire addressing demographics, previous SARS-CoV-2 infections, COVID-19 vaccinations, and 37 symptoms (grouped into 10 categories including cardiovascular complaints, exercise capacity, respiratory tract, and ears/nose/throat [ENT]; also see Table 2) compatible with PCS in the previous three months outside of other acute infections (e.g., common cold). For the symptom questionnaire, the DGKJ (German Society for Paediatrics and Adolescent Medicine) questionnaire for the “standardised primary care of children and adolescents with long COVID” was used, supplemented by innovative questions and questions previously used in the longitudinal study of one of the participating cohorts [22]. The intensity and degree of impairment in the activities of daily living were assessed for each symptom reported above. General health, quality of life (QoL), and mental state were assessed using a WHO-standardised health-related questionnaire [23–27], with five descriptors that were converted into a nominal scale (− 2 = most negative to + 2 = most positive).

The number of symptoms reported by participants was used to construct a variable with three categories (none, few [≤ 4], or many [> 4] symptoms). A composite variable for “physical performance” was subsequently set to “yes” if fitness was assessed as “impaired” or “lost” or if ≥ 1 of the following 4 symptom(s) was reported (“dyspnoea on exertion”, “impaired physical capacity”, “circulatory problems on exertion”).

Descriptive statistics (number and percentage, *n* [%]) were used to compare the groups. Differences in the frequency of nominal data between the two groups were tested for significance using the chi-squared test, and the respective odds ratio (OR) and corresponding 95% confidence interval (CI) were calculated for individual symptoms. The influence of age, sex, SARS-CoV-2 status, and vaccination status on the prevalence of persistent symptoms was tested using logistic regression analysis. Differences with *p* < 0.05 were considered to be statistically significant. Month and year of birth were recorded in the interview, and, for all calculations of time intervals (e.g., time after infection), the 15th day of the respective month was used.

The study was approved by the local Ethics Committees of Bochum (reference BO-20/6927_7) and Dresden (reference BO-EK-156042020) and was conducted in accordance with the Declaration of Helsinki. All children and adolescents provided verbal, and all guardians provided written informed consent to participate in the study.

## Results

### Patient characteristics

**Fig. 1:**
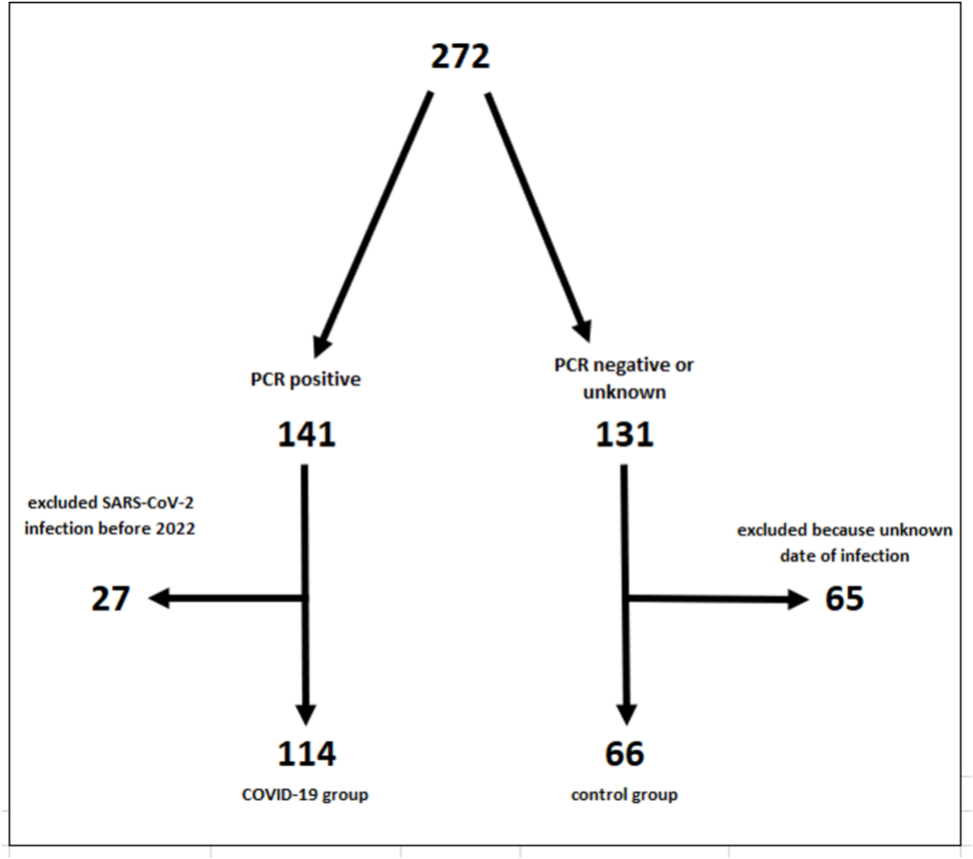
Flow chart of study enrolment

A total of 272 children and adolescents were recruited as part of the “Immunebridge” project (Dresden [*n* = 108], Bochum [*n* = 164]). Of these, 92 were excluded due to SARS-CoV-2 infection before 2022 (n = 27) and positive N-AK titre but an unknown point in time of infection (*n* = 65) (Fig. 1). Of the remaining participants, 114 children and adolescents had SARS-CoV-2 infection confirmed by PCR testing in 2022 (COVID-19 group), which could be confirmed in 108 (94.7%) participants by N-AK detection. Sixty-six children and adolescents with a PCR-negative history of SARS-CoV-2 infection and seronegativity for SARS-CoV-2 N-AK served as the control group.

Aside from a higher proportion of adolescents and associated higher vaccination rate in the control group (66.7% adolescents, 89.4% vaccinated; both p < 0.01), than in the COVID-19 group (49.1% adolescents, 61.4% vaccinated), the groups hardly differed from one another (Table 1).

**Table 1:** Characteristics of all study participants, the COVID-19 group and the control group

		All	COVID-19 group	Control group
**Number**		**180**	**114**	**66**
**Sex**	Female	**105 (58.3%)**	**66 (57.9%)**	**39 (59.1%)**
**Age**^**#**^	Years	**12.24 (± 4.16)**	**11,65 (± 3,82)**	**13.08 (± 4.45)**
	5–6 years	**30 (16.7%)**	**16 (14%)**	**14 (21.2%)**
	7–11 years	**50 (27.8%)**	**42 (36.8%)**	**8 (12.1%)**
	12–18 years	**100 (55.6%)**	**56 (49.1%)**	**44 (66.7%)**
Immigration background*		**178**	**114**	**64**
	Yes	**8 (4.5%)**	**6 (5.3%)**	**2 (3.1%)**
	One parent	**13 (7.3%)**	**8 (7%)**	**5 (7.8%)**
	Missing	**2 (1.1%)**	**0 (0%)**	**2 (3.1%)**
Education of parents*		**175**	**113**	**62**
	A-levels (higher education)	**132 (75.4%)**	**85 (75.2%)**	**47 (75.8%)**
	Missing	**5 (2.8%)**	**1 (0.9%)**	**4 (6.3%)**
**COVID-19 vaccination**^**#**^				
	Yes	**129 (71.7%)**	**70 (61.4%)**	**59 (89.4%)**
	No	**49 (27.2%)**	**42 (36.8%)**	**7 (10.6%)**
	Missing	**1 (0.6%)**	**1 (0.9%)**	**0 (0%)**
**SARS-CoV-2 antibodies**				
	N-AK positive	**108 (60%)**	**108 (94.7%)**	**0 (0%)**
	S-AK positive	**172 (95.6%)**	**113 (99.1%)**	**59 (89.4%)**

(*number of valid responses, ^**#**^statistically significant difference between the two groups, *p* < 0.01).

### Frequency of typical PCS symptoms

Of the COVID-19 group, 64.9% reported ≥ 1 PCS-associated symptom(s), which corresponded with the proportion of children in the control group (62.1%). Reported PCS-associated symptoms in the COVID-19 and control groups are summarised in Table 2. Only concentration disorders were significantly more prevalent in the COVID-19 group than in the control group (12.3% vs. 1.5%, respectively; *p* = 0.012). All other symptoms occurred with equal frequency in both groups.

**Table 2:** Number (n (%)) of children and adolescents in the total cohort, the COVID-19 group, and the control group suffering from typical PCS symptoms during the observation period, divided into different organ systems and individual symptoms with indication of odds ratio (OR) and 95% confidence interval (95% CI)

	All	COVID-19 group	Control group	OR (95%CI)
**Cardiovascular complaints**	21 (11.7%)	15 (13.2%)	6 (9.1%)	1.52 (0.56…4.12)
Low impact on everyday functioning	9 (5%)	6 (5.3%)	3 (4.5%)	1.17 (0.28…4.83)
High impact on everyday functioning	7 (3.9%)	5 (4.4%)	2 (3%)	1.47 (0.28…7.79)
Palpitations	7 (3.9%)	4 (3.5%)	3 (4.5%)	0.76 (0.17…3.52)
Chest pain	8 (4.4%)	7 (6.1%)	1 (1.5%)	4.25 (0.51…35.35)
Circulatory problems at rest	7 (3.9%)	4 (3.5%)	3 (4.5%)	0.76 (0.17…3.52)
Circulatory problems during exertion*	10 (5.6%)	8 (7%)	2 (3%)	2.42 (0.5…11.73)
Faint	0 (0%)	0 (0%)	0 (0%)	
**Exercise capacity**	29 (16.1%)	22 (19.3%)	7 (10.6%)	2.02 (0.81…5.01)
Low impact on everyday functioning	14 (7.8%)	10 (8.8%)	4 (6.1%)	1.49 (0.45…4.96)
High impact on everyday functioning	14 (7.8%)	11 (9.6%)	3 (4.5%)	2.24 (0.6…8.35)
Exhaustion	26 (14.4%)	19 (16.7%)	7 (10.6%)	
Limited physical capacity*	13 (7.2%)	11 (9.6%)	2 (3%)	
**Respiratory complaints and ENT**	33 (18.3%)	21 (18.4%)	12 (18.2%)	1.02 (0.46…2.23)
Low impact on everyday functioning	19 (10.6%)	11 (9.6%)	8 (12.1%)	0.77 (0.29…2.03)
High impact on everyday functioning	7 (3.9%)	6 (5.3%)	1 (1.5%)	3.61 (0.43…30.67)
Dyspnoea at rest	3 (1.7%)	3 (2.6%)	0 (0%)	
Dyspnoea during exertion*	15 (8.3%)	13 (11.4%)	2 (3%)	
Cough, sneezing, sore throat, earache	24 (13.3%)	13 (11.4%)	11 (16.7%)	
**Gustatory and olfactory complaints**	8 (4.4%)	6 (5.3%)	2 (3%)	1.78 (0.35…9.07)
Low impact on everyday functioning	7 (3.9%)	5 (4.4%)	2 (3%)	1.47 (0.28…7.79)
high impact on everyday functioning	1 (0.6%)	1 (0.9%)	0 (0%)	
Olfactory disorder	2 (1.1%)	2 (1.8%)	0 (0%)	
Taste disorders	2 (1.1%)	2 (1.8%)	0 (0%)	
New aversion to certain foods	6 (3.3%)	4 (3.5%)	2 (3%)	
**Fever**	10 (5.6%)	8 (7%)	2 (3%)	2.42 (0.5…11.73)
Low impact on everyday functioning	2 (1.1%)	2 (1.8%)	0 (0%)	
High impact on everyday functioning	6 (3.3%)	5 (4.4%)	1 (1.5%)	2.98 (0.34…26.09)
Fever > 38.5°Celsius	9 (5%)	8 (7%)	1 (1.5%)	
Chills	2 (1.1%)	1 (0.9%)	1 (1.5%)	
**Menstrual cramps**	16 (8.9%)	8 (7%)	8 (12.1%)	0.55 (0.2…1.53)
Low impact on everyday functioning	12 (6.7%)	7 (6.1%)	5 (7.6%)	0.8 (0.24…2.62)
High impact on everyday functioning	3 (1.7%)	1 (0.9%)	2 (3%)	0.28 (0.03…3.18)
**Gastrointestinal complaints**	54 (30%)	38 (33.3%)	16 (24.2%)	1.56 (0.79…3.1)
Low impact on everyday functioning	30 (16.7%)	19 (16.7%)	11 (16.7%)	1 (0.44…2.26)
High impact on everyday functioning	13 (7.2%)	10 (8.8%)	3 (4.5%)	2.02 (0.54…7.62)
Loss of appetite	7 (3.9%)	4 (3.5%)	3 (4.5%)	
Weight gain or weight loss	3 (1.7%)	3 (2.6%)	0 (0%)	
Abdominal pain	36 (20%)	27 (23.7%)	9 (13.6%)	1.97 (0.86…4.49)
Nausea and/or vomiting	15 (8.3%)	10 (8.8%)	5 (7.6%)	
Diarrhoea	19 (10.6%)	11 (9.6%)	8 (12.1%)	
**Complaints of musculoskeletal system and skin**	31 (17.2%)	20 (17.5%)	11 (16.7%)	1.06 (0.47…2.39)
Low impact on everyday functioning	15 (8.3%)	10 (8.8%)	5 (7.6%)	1.17 (0.38…3.59)
High impact on everyday functioning	7 (3.9%)	5 (4.4%)	2 (3%)	1.47 (0.28…7.79)
Muscle pain and/or weakness	15 (8.3%)	11 (9.6%)	4 (6.1%)	
Joint pain and/or joint swelling	14 (7.8%)	9 (7.9%)	5 (7.6%)	
Skin rash	14 (7.8%)	7 (6.1%)	7 (10.6%)	
blue/reddened fingers/toes	1 (0.6%)	0 (0%)	1 (1.5%)	
**Headache and neurological complaints**	63 (35%)	40 (35.1%)	23 (34.8%)	1.01 (0.54…1.91)
Low impact on everyday functioning	32 (17.8%)	21 (18.4%)	11 (16.7%)	1.13 (0.51…2.52)
High impact on everyday functioning	21 (11.7%)	16 (14%)	5 (7.6%)	1.99 (0.69…5.71)
Headache	44 (24.4%)	28 (24.6%)	16 (24.2%)	1.02 (0.5…2.06)
Dizziness	18 (10%)	11 (9.6%)	7 (10.6%)	
Tingling or pain in arms/legs	2 (1.1%)	2 (1.8%)	0 (0%)	
Local deafness/paralysis	5 (2.8%)	0 (0%)	5 (7.6%)	
Concentrations disorders	15 (8.3%)	14 (12.3%)	1 (1.5%)	**9.1 (1.17…70.88)**^**#**^
Memory impairment/learning problems	14 (7.8%)	11 (9.6%)	3 (4.5%)	
Sleep disorders	13 (7.2%)	9 (7.9%)	4 (6.1%)	
**Psychological complaints**	28 (15.6%)	19 (16.7%)	9 (13.6%)	1.27 (0.54…2.99)
Low impact on everyday functioning	16 (8.9%)	12 (10.5%)	4 (6.1%)	1.82 (0.56…5.9)
High impact on everyday functioning	8 (4.4%)	6 (5.3%)	2 (3%)	1.78 (0.35…9.07)
Aggression	10 (5.6%)	6 (5.3%)	4 (6.1%)	0.86 (0.23…3.17)
Hyperactivity	9 (5%)	7 (6.1%)	2 (3%)	2.09 (0.42…10.39)
Anxiety	13 (7.2%)	9 (7.9%)	4 (6.1%)	1.33 (0.39…4.5)
Sadness/depression	12 (6.7%)	8 (7%)	4 (6.1%)	1.17 (0.34…4.04)
**Other complaints**	8 (4.4%)	3 (2.6%)	5 (7.6%)	0.33 (0.08…1.43)
**Physical performance**^**§**^				**3.08 (1.33…7.15)**^**#**^

^§^The score “physical performance” was subsequently formed in a post hoc analysis from parameters marked with “*”, and the parameter “assessment of own fitness” in Table 4

^#^Statistical significant difference (*p* < 0.05)

### Variety and severity of typical PCS symptoms

There were no differences in the number of symptoms per child: 45.6% of the children and adolescents in the COVID-19 group experienced only a few symptoms (≤ 4) compared with 47% in the control group. Multiple symptoms (> 4) were reported slightly more frequently by those in the COVID-19 group (19.3%) compared with 15.2% in the control group, although the difference was not statistically significant (OR 1.34 [95% CI 0.59–3.03]) (Table 3). Children and adolescents in both groups were equally affected in their activities of daily living (64.9% vs. 62.1%). Children in the COVID-19 group were more frequently affected in multiple organ systems than in the control group (15.8% vs. 9.7%, respectively), which did not reach statistical significance (OR 1.88 [95% CI 0.7–4.99]) (Table 3).

**Table 3:** Number (*n* (%)) of children and adolescents with none, less or more than four typical PCS symptoms, and number of children whose daily functions were impaired by these typical PCS symptoms divided according to the number of organ systems affected in the total cohort, the COVID-19 group, and the control group with indication of odds ratio (OR) and 95% confidence interval (95% CI)

	All	COVID-19 group	Control group	OR (95%CI)
Number of children and adolescents with PCS typical symptoms				
None	**65 (36.1%)**	**40 (35.1%)**	**25 (37.9%)**	0.89 (0.47…1.66)
≤ 4 symptoms	**83 (46.1%)**	**52 (45.6%)**	**31 (47%)**	0.95 (0.52…1.74)
> 4 symptoms	**32 (17.8%)**	**22 (19.3%)**	**10 (15.2%)**	1.34 (0.59…3.03)
Number of children whose daily functions were impaired by the PCS-typical symptoms divided according to the number of organ systems affected				
1–2 organ systems	**91 (50.6%)**	**56 (49.1%)**	**35 (53%)**	0.86 (0.47…1.57)
3–6 organ systems	**24 (13.3%)**	**18 (15.8%)**	**6 (9.1%)**	1.88 (0.7…4.99)

### Patterns of typical PCS symptoms

**Fig. 2:**
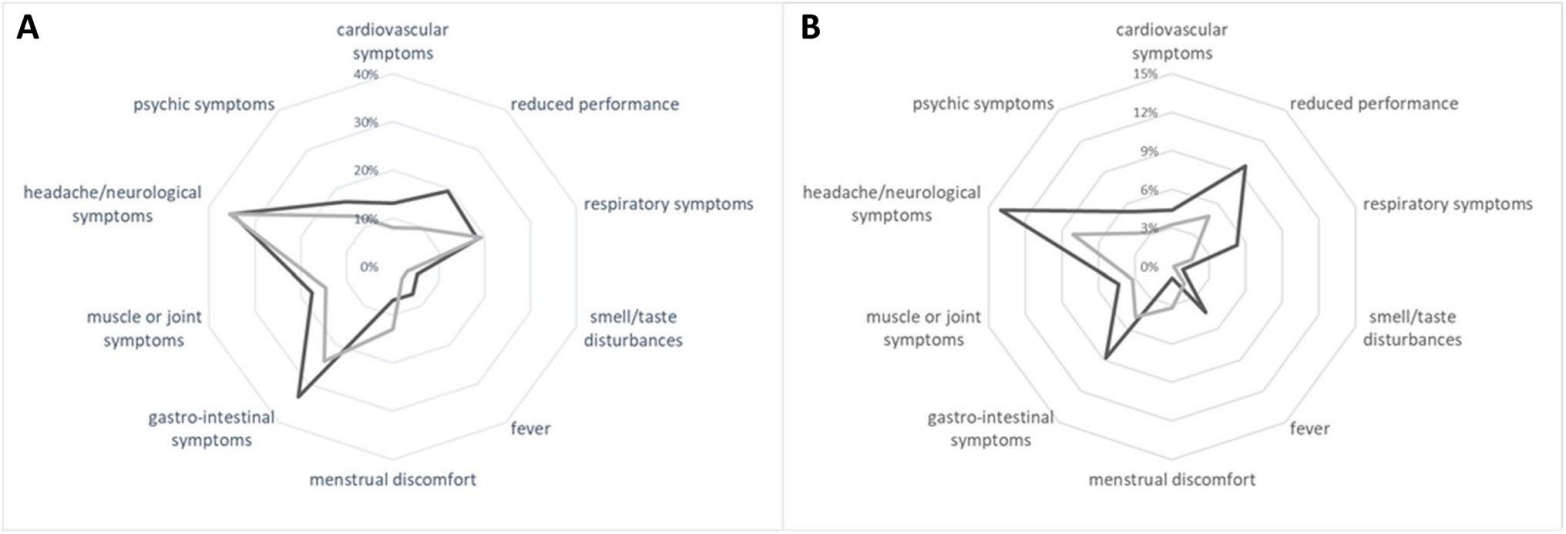
**A, B** Distribution of PCD-typical symptoms (**A**) and severe impairment of everyday function by these symptoms (**B**) divided by organ systems between the COVID-19 group (black line) and the control group (grey line).

When comparing the distribution of symptoms according to organ system, the pattern of symptoms was found to be very similar between the COVID-19 and control groups (Fig. 2A). Children and adolescents in the COVID-19 group were more likely to report severe impairment of daily functioning in some organ systems, although this did not reach statistical significance (Fig. 2B).

The analysis of the score “physical performance” (i.e. derived from the parameters “dyspnoea during exertion”, “limited physical capacity”, “circulatory problems during exertion”, and “assessment of own fitness”) revealed a significant impaired physical performance (OR 3.08 [95% CI 1.33–7.15]; *p* = 0.016) in the COVID-19 group. Univariate analysis of variance confirmed this significant difference, regardless of age, sex, and vaccination status.

### Age dependency of typical PCS symptoms

**Fig. 3:**
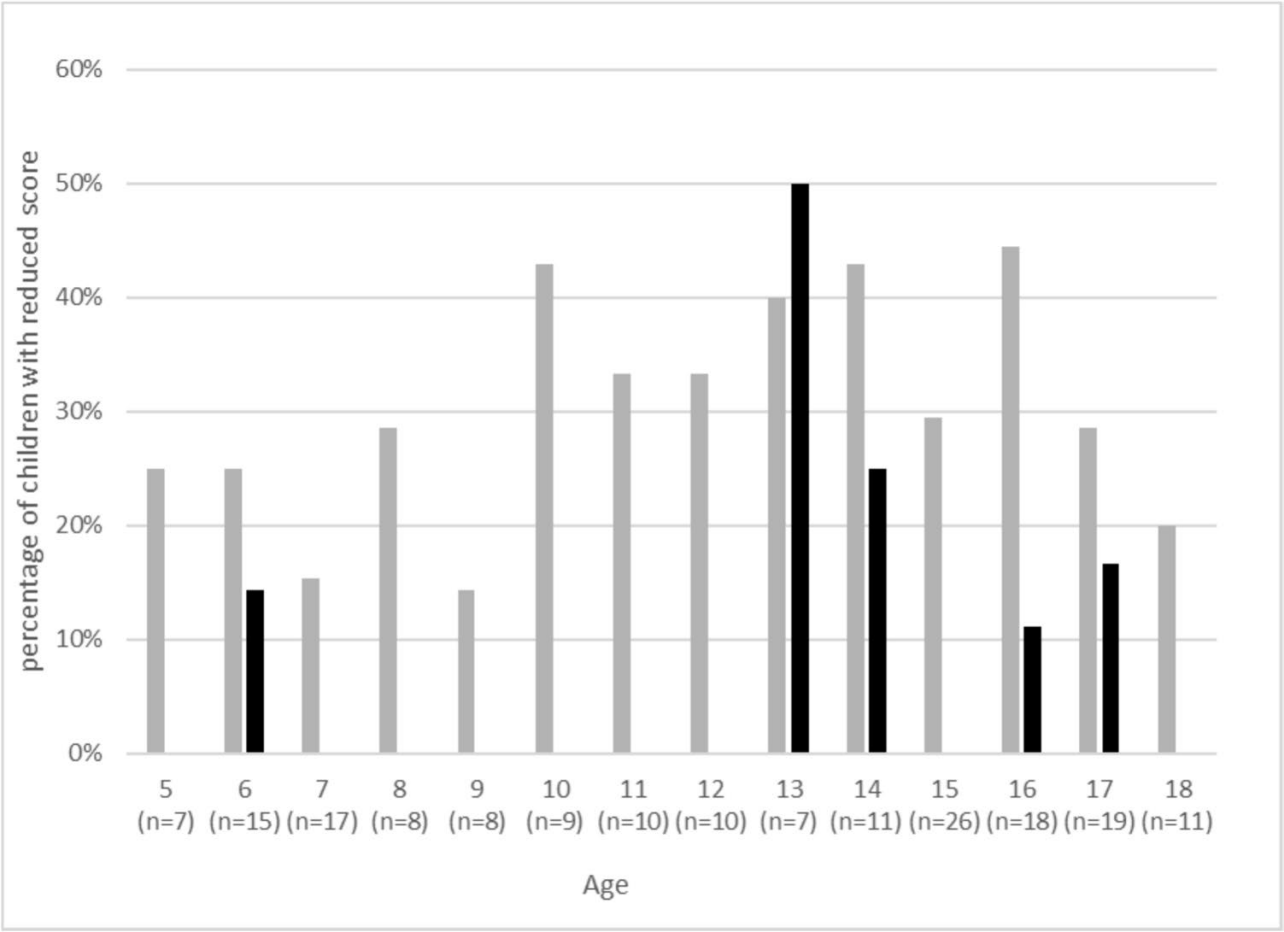
Frequency of a reduced score “physical performance” in different age groups in the COVID-19 group (grey columns) and the control group (black columns). *X*-axis: age in years and number of total participants with this age (*n* = x). *Y*-axis: percentage of children in this age group with a reduced score “physical performance”. The score “physical performance” was subsequently formed in a post hoc analysis from the parameters “dyspnoea on exertion”, “impaired physical capacity”, and “circulatory problems on exertion” in the questionnaire (Table 2) and the parameter “assessment of own fitness” (Table 4)

**Fig. 4:**
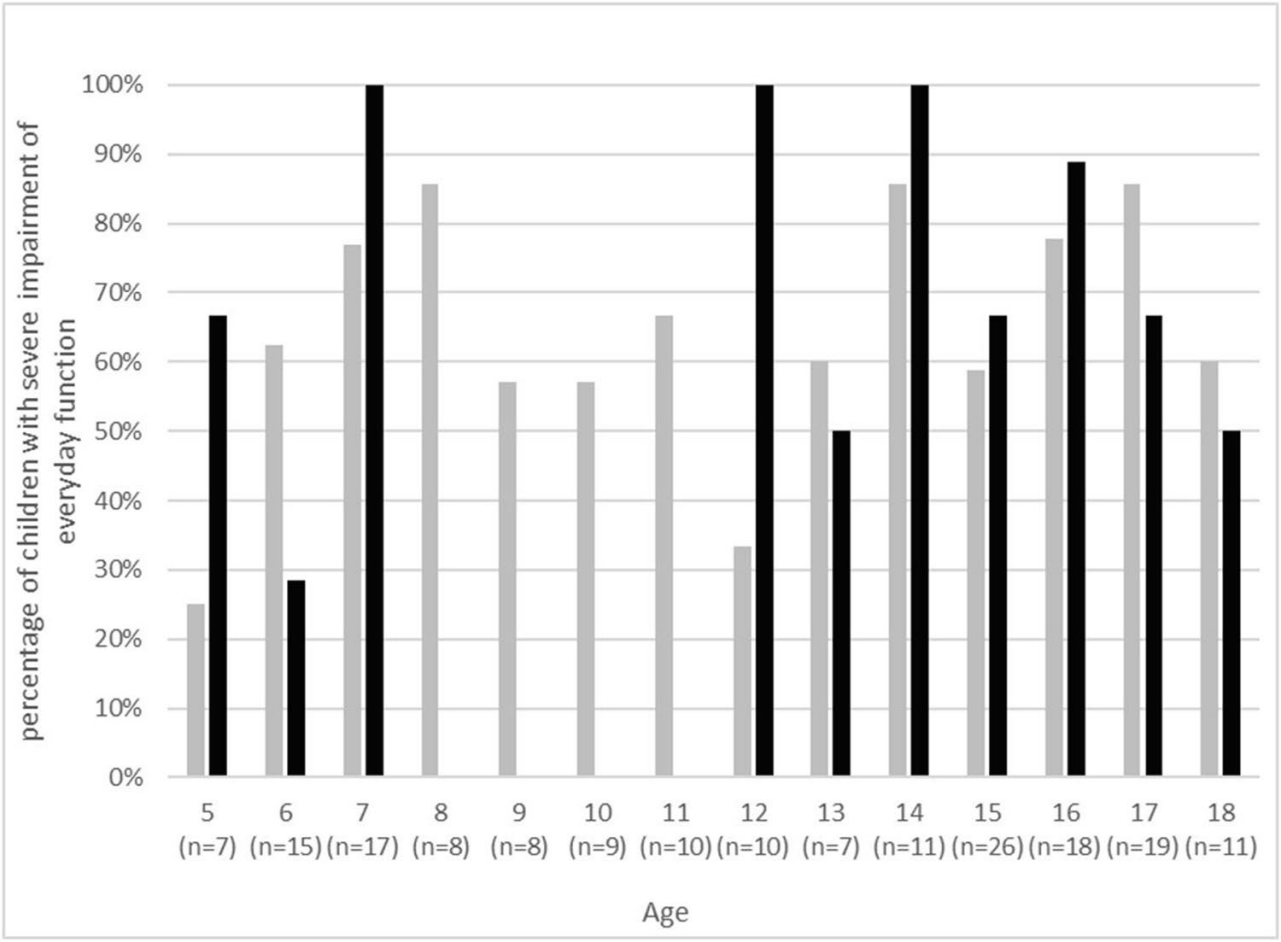
Proportion of children in different age groups who reported severe impairment of their everyday function due to PCS-typical symptoms in the COVID-19 group (grey columns) and the control group (black columns). *X-*axis: age in years and number of total participants with this age (*n* = x). *Y*-axis: percentage of children in this age group reporting severe impairment of their everyday function due to PCS-typical symptoms

In the COVID-19 group, symptoms associated with PCS were reported at all ages, in contrast to the control group, in which symptoms were mainly reported in the youngest age group and by adolescents (Figs. 3 and 4).

### QoL

Eighty-seven percent of the children and adolescents in both groups rated their QoL as excellent or very good (Table 4). There were no significant differences between the COVID-19 and control groups in terms of QoL, general health, or mental state. However, children and adolescents were less likely to feel physically fit after COVID-19 compared with controls, narrowly failing to reach statistical significance (OR 4.48 [95% CI 0.99–20.37]). Comparing girls and boys after COVID-19, a trend demonstrated that girls > 11 years of age rated their physical performance and mental state as worse. However, this trend was not observed in the control group (Online Supplement, Fig. 1).

**Table 4:** Responses to questions about health, quality of life, physical performance, and mental state in the total cohort, the COVID-19 group, and the control group with indication of odds ratio (OR) and 95% confidence interval (95% CI)

	All	COVID-19 group	Control group	OR (95%CI)
***N***	180	114	66	
General health*	179	114	65	
Excellent	66 (36.9%)	38 (33.3%)	28 (43.1%)	0.68 (0.36…1.27)
Very good	84 (46.9%)	55 (48.2%)	29 (44.6%)	1.19 (0.65…2.19)
Good	28 (15.6%)	21 (18.4%)	7 (10.8%)	1.9 (0.76…4.75)
Less good or bad	1 (0.6%)	0 (0%)	1 (1.5%)	-
Quality of life*	177	113	64	
Excellent	68 (38.4%)	44 (38.9%)	24 (37.5%)	1.1 (0.59…2.06)
Very good	87 (49.2%)	55 (48.7%)	32 (50%)	0.99 (0.54…1.82)
Good	21 (11.9%)	13 (11.5%)	8 (12.5%)	0.93 (0.37…2.38)
Less good or bad	1 (0.6%)	1 (0.9%)	0 (0%)	-
Fitness*	178	114	64	
Very	69 (38.8%)	39 (34.2%)	30 (46.9%)	0.62 (0.34…1.16)
Good	79 (44.4%)	52 (45.6%)	27 (42.2%)	1.21 (0.66…2.24)
Moderate	14 (7.9%)	9 (7.9%)	5 (7.8%)	1.05 (0.34…3.26)
Reduced or loss	16 (9%)	14 (12.3%)	2 (3.1%)	4.48 (0.99…20.37)
Full of energy*	178	114	64	
Always	58 (32.6%)	37 (32.5%)	21 (32.8%)	1.03 (0.54…1.97)
Often	89 (50%)	56 (49.1%)	33 (51.6%)	0.97 (0.53…1.77)
Sometimes	28 (15.7%)	18 (15.8%)	10 (15.6%)	1.05 (0.45…2.43)
Rare or never	3 (1.7%)	3 (2.6%)	0 (0%)	-
Mental feeling*	178	114	64	
Excellent	43 (24.2%)	28 (24.6%)	15 (23.4%)	1.11 (0.54…2.27)
Very good	75 (42.1%)	44 (38.6%)	31 (48.4%)	0.71 (0.38…1.31)
Good	52 (29.2%)	35 (30.7%)	17 (26.6%)	1.28 (0.65…2.52)
Less good or bad	8 (4.5%)	7 (6.1%)	1 (1.6%)	4.25 (0.51…35.35)
Sadness*	178	114	64	
Always or often	4 (2.2%)	4 (3.5%)	0 (0%)	-
Sometimes	36 (20.2%)	26 (22.8%)	10 (15.6%)	1.65 (0.74…3.69)
Rare	81 (45.5%)	45 (39.5%)	36 (56.3%)	0.54 (0.29…1)
Never	57 (32%)	39 (34.2%)	18 (28.1%)	1.39 (0.71…2.7)
Loneliness*	178	114	64	
Always or often	1 (0.6%)	1 (0.9%)	0 (0%)	-
Sometimes	12 (6.7%)	6 (5.3%)	6 (9.4%)	0.56 (0.17…1.8)
Rare	49 (27.5%)	31 (27.2%)	18 (28.1%)	1 (0.5…1.97)
Never	116 (65.2%)	76 (66.7%)	40 (62.5%)	1.3 (0.69…2.44)

Both negative responses are combined in each case (* number of valid responses)).

## Discussion

We evaluated the frequency of PCS-related symptoms in children and adolescents after SARS-CoV-2 infection in the Omicron era compared with SARS-CoV-2-seronegative controls. Despite the increase in SARS-CoV-2 infections during the Omicron wave, the number of children with ≥ 1 typical PCS symptom(s) was similar in both groups, as was the frequency of the symptoms reported individually. However, impaired concentration and reduced physical performance were significantly more common among children with Omicron infection. Children in the COVID-19 group rated their fitness as worse, with otherwise equal ratingsSSS of QoL regarding general and mental health.

According to a recently published study comparing typical PCS symptoms in children across different pandemic waves [17], the relative number of children and adolescents with PCS-like symptoms (64.9%) in our COVID-19-cohort was of the same magnitude as in COVID-negative controls (62.1%) during the Omicron wave. In contrast, in our cohort, the proportion of children reporting typical PCS symptoms following SARS-CoV-2 infection was similar to that reported during the SARS-CoV-2 Alpha wave. Conducted in the UK, the Children and Young People with Long COVID (CLoCk) study reported 66.5% of 3065 adolescents with persistent symptoms 3 months after SARS-CoV-2 infection compared with 53.3% of healthy controls [2]. In a large Danish cohort study, Borch et al. [28] reported a prevalence of up to 51% in children and adolescents versus up to 38% in controls > 4 weeks after SARS-CoV-2 infection.

Consistent with other studies, we found a high burden of symptoms in both patients and controls, pointing toward factors other than infection contributing to PCS-like symptoms. Therefore, we suspect that pandemic-related interventions will have a major impact on children’s physical and mental health. This assumption is supported by the measurable decline in the physical and mental health and QoL of children and adolescents [29–32]. Contributing factors may include school closures and related restrictions on the lives of children and young individuals. Despite a slight improvement, the rate of reported health problems remained above pre-pandemic levels [29, 33, 34].

Another explanation for the higher burden of complaints in the control group may be the slightly higher proportion of adolescents in our control group compared with the COVID-19 group (66.7% vs. 49.1%, respectively). Data regarding high prevalence of symptoms, which were also grouped as “PCS-like”, already existed in this age group before the pandemic. For example, > 20–30% of adolescents experience fatigue [35, 36]. In a representative German study (KIGGS cohort) in 2019, 45.2% of 11–17-year-old girls reported recurrent headaches, recurrent abdominal pain (34.5%), and recurrent back pain (28.3%) [37]. This high burden of symptoms among adolescents independent of COVID-19 was also observed in our study cohort. For example, two-thirds of the participants in the control group who reported reduced physical performance were > 12 years of age (Fig. 3). Regarding the sex association, our study revealed a trend, rather than a confirmed higher symptom burden after COVID-19, among girls and adolescents [2, 5]. Unfortunately, the literature lacks pre-pandemic prevalence data for all PCS symptoms with which to compare our cohort. Our findings highlight the need for more robust longitudinal research to track symptom prevalence in the future.

Despite the high frequency and burden of reported typical PCS symptoms in the COVID-19 and control groups, children and adolescents rated their QoL and general and mental health mostly as excellent or very good. We found no significant differences between the two groups, which is consistent with findings from other larger cohorts [2, 7]. Thus, the decline in QoL observed in children and adolescents during the pandemic [29, 30] appears to be independent of SARS-CoV-2 infection. However, children and adolescents rated their fitness as worse if they experienced SARS-CoV-2 infection than the control group.

Compared with the initial years of the pandemic, the incidence of PCS decreased significantly in the Omicron era [14, 15, 17]. However, following infection with the Omicron variant, PCS-associated symptoms, such as fatigue and dermatological, gastrointestinal, sleep, and sensory manifestations, have also been described in children and adolescents [15–17]. However, in accordance with the study by Pazukhina et al. [17], our analysis of a non-selected cohort compared with a control group did not confirm that many of these symptoms were purely SARS-CoV-2 related, although we also observed significantly impaired physical performance in the COVID-19 group. Of note, olfactory and gustatory disturbances were rarely reported in our cohort, which is still one of the most common symptoms reported in the Alpha wave [28] and is listed as a major symptom according to the WHO definition [3].

### Limitations and strengths

Limitations of our study include its retrospective design and relatively small sample size. Unfortunately, the time interval between infection and study enrolment was not recorded for all participants. In the majority of the children in Bochum, the infection had occurred more than 6 months previously. Because of the uncertainty in the other participants, we avoided using the term confirmed PCS and only analysed the frequency of PCS-associated symptoms. The study relied on self- or parent-reported symptoms in the absence of clinical assessment. Parents answering questions on behalf of their children can lead to higher or lower levels of complaints, and knowledge of Sars-CoV-2 infection could have led to false-positive results. Although we recruited from a non-selective cohort, we cannot exclude the possibility that families who were very sensitive to PCS-associated symptoms enrolled in the study. This could be another explanation for the high symptom rates in the COVID-19 and control groups.

Strengths of our study included the analysis of a non-selective cohort of children and adolescents after SARS-CoV-2 infection compared with a control group, and the strict inclusion criteria of PCR-confirmed SARS-CoV-2 infection and antibody analysis, which are among the main points of criticism in other PCS studies [18].

## Conclusion

Children with and without previous infection with SARS-CoV-2 Omicron did not differ in most PCS-associated symptoms. The only exceptions were physical performance and mental and cognitive problems, which appeared to be more impaired in the long term after SARS-CoV-2 Omicron infection than in control children. These aspects should warrant special attention in PCS-related healthcare diagnoses and treatments.

## Collaborators

IMMUNEBRIDGE KIDS study group: Lynn Eitner, Geraldine Engels, Uta Falke, Anna Hoffmann, Sarah Holzwarth, Johannes Liese, Josephine Schneider, Michaela Schwarzbach, Andrea Streng.

IMMUNEBRIDGE Study Group: Antonia Bartz, Sabine Blaschke-Steinbrecher, Gunnar Brandhorst, Melanie Brinkmann, Kathrin Budde, Axel Budde, Marek Deckena, Maren Dreier, Marc Fenzlaff, Johannes Forster, Christoph Härtel, Manuela Harries, Max Hassenstein, Peter Heuschmann, Elena Hick, Katharina Hecker, Isabell von Holt, Olga Hovardovska, Veronika Jäger, Andre Karch, Katja Kehl, Mirjam Kohls, Rüdiger van Kries, Stefan Krüger, Marc-André Kurosinski, Oliver Kurzai, Berit Lange, Kristin Meyer-Schlinkmann, Matthias Nauck, Anna-Lisa Oechsle, Patrick Ottensmeyer, Franziska Pietsch, Jens-Peter Reese, Daniel Rosenkranz, Nicole Rübsamen, Viktoria Rücker, Mario Schattschneider, Christin Schäfer, Simon Schlinkert, Horst Schroten, Hendrik Streeck, Kai Schulze-Wundling, Stefan Störk, Carsten Tiemann, Henry Völzke, Theresa Winter.

## Supplementary information

Below is the link to the electronic supplementary material.

Supplementary file1 (DOCX 55 KB)

## Data Availability

The data that support the findings of this study are available from the corresponding author upon reasonable request.

## Abbreviations

AK: Antibodies
BAU: Binding antibody units
CI: Confidence interval
CLoCk: Children and Young People with Long COVID
COI: Assay-specific cut-off index
COVID-19: Post-coronavirus disease 2019
ENT: Ears/nose/throat
Long COVID: Long coronavirus disease-2019
MIS-C: Multisystem inflammatory syndrome in children
N-AK: Antibodies against the nucleocapsid protein
NUM: German Network University Medicine
OR: Odds ratio
PCR: Polymerase chain reaction
PCS: Post-coronavirus disease 2019 syndrome
QoL: Quality of life
S-AK: Antibodies against the SARS-CoV-2 spike protein
SARS-CoV-2: Severe acute respiratory syndromecoronavirus type 2
WHO: World Health Organisation
