# Supplementary material for: Post-coronavirus disease 2019–associated symptoms among children and adolescents in the SARS-CoV-2 Omicron era
Source: Eur J Pediatr. 2024 Dec 21;184(1):96. doi: 10.1007/s00431-024-05919-3 (PMC11662056; doi:10.1007/s00431-024-05919-3)
Supplement: Supplementary file 1 — Supplementary file1 (DOCX 55 KB) [file 431_2024_5919_MOESM1_ESM.docx]

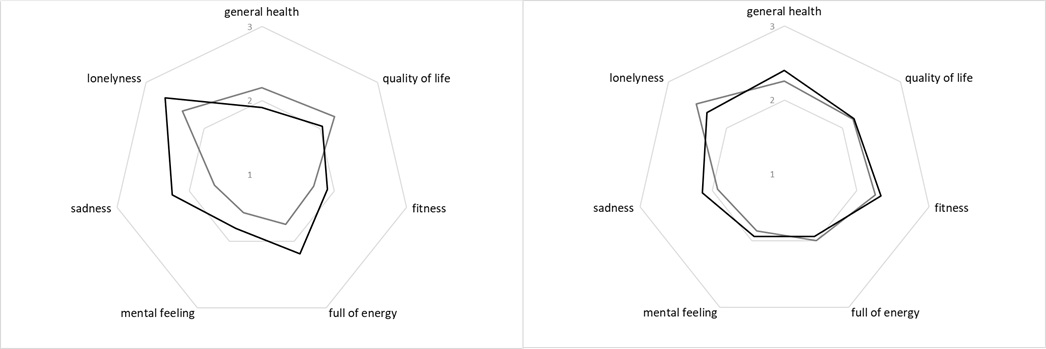


**Online Supplement Figure 1:**

Assessment of own Quality of Life of girls (grey line) and boys (black line) over 11 years of age in the COVID-19 group (left) and the control group (right). In the evaluation of the questions on well-being and QoL (see Table 3), the 5 answer categories were converted into a nominal scale (-2 to +2) for graphical representation, whereby the most positive answer was coded with +2 and the most negative answer with -2. 1-3 are the result of the calculation of this score.
